# Genome Sequencing and Comparative Transcriptomic Analysis of Rice Brown Spot Pathogen *Bipolaris oryzae* Adaptation to Osmotic Stress

**DOI:** 10.3390/jof11030227

**Published:** 2025-03-17

**Authors:** Chun Wang, Kexin Yang, Sauban Musa Jibril, Ruoping Wang, Chengyun Li, Yi Wang

**Affiliations:** 1 State Key Laboratory for Conservation and Utilization of Bio-Resources in Yunnan, Yunnan Agricultural University, Kunming 650201, China; chunwang775@gmail.com (C.W.); 18468006252@163.com (K.Y.); saubanzango@gmail.com (S.M.J.); 19885472238@163.com (R.W.); 2 Yunnan-CABI Joint Laboratory for Integrated Prevention and Control of Transboundary Pests, Yunnan Agricultural University, Kunming 650201, China

**Keywords:** rice brown spot, single-molecule real-time sequencing, transcriptome analysis, osmotic stress

## Abstract

Rice brown spot disease, caused by *Bipolaris oryzae*, is a significant fungal disease that poses a major threat to global rice production. Despite its widespread impact, genomic studies of *B. oryzae* remain limited, particularly those involving high-quality genomic data. In this study, we performed whole-genome sequencing of the *B. oryzae* strain RBD1, which was isolated from the demonstration field for upland rice cultivation in Haozhiba Village, Lancang County, Pu’er City, Yunnan Province, China, using a combination of second-generation Illumina sequencing and third-generation Single-Molecule Real-Time (SMRT) sequencing. The assembled genome was 37.5 Mb in size with a G + C content of 49.39%, containing 42 contigs with a contig N50 of 2.0 Mb. Genomic analysis identified genes related to carbon, nitrogen, and lipid metabolism, highlighting the strain’s metabolic flexibility under diverse environmental conditions and host interactions. Additionally, we identified pathogenicity-related genes involved in MAPK signaling, G protein signaling, and oxidative stress responses. Under 1.2 M sorbitol-induced osmotic stress, we observed significant differences in growth responses between RBD1 and the rice blast fungus *Magnaporthe oryzae* H7. Transcriptomic analysis using Illumina sequencing revealed that RBD1 responds to osmotic stress by enhancing carbohydrate metabolism, fatty acid degradation, and amino acid synthesis, while H7 primarily relies on protein synthesis to enhance growth tolerance. This study provides a valuable foundation for understanding the pathogenic mechanisms of rice brown spot and future disease control strategies.

## 1. Introduction

Rice brown spot, caused by *Bipolaris oryzae* (Breda de Haan) Shoemaker, was first documented in Japan in 1900. Initially referred to as “nai-yake”, “sesame leaf spot”, or “Helminthosporiosis”, it has since become a globally significant rice disease. The severity of outbreaks varies across different rice-growing regions worldwide. For example, in 1942, a severe epidemic in Bangladesh triggered a devastating famine [1]. In recent years, large-scale outbreaks have been reported in China. In 2004, an outbreak in Yongning, Guangxi Province, affected approximately 10,000 hectares. By 2008, the affected area in Zhaoping, Guangxi, had reached 4500 hectares, resulting in a rice yield loss exceeding 10.12 million kg [2].

Rice brown spot can occur throughout the entire growth cycle of rice, from the seedling stage to harvest. While the disease primarily infects rice leaves, it can also affect the leaf sheaths, panicles, and grains. During the seedling stage, infected coleoptiles turn brown, and in severe cases, the seedlings may wither after the sheath emerges. At the mature stage, brown, oval to nearly circular lesions appear on the leaves. These lesions may expand and develop concentric rings, impairing photosynthesis and eventually causing plant wilting and death. Infection of the panicles can disrupt grain filling, leading to the formation of empty grains. The spread and severity of rice brown spot are closely linked to environmental conditions, including soil silicon content, temperature, and humidity. Low soil silicon levels increase disease incidence, while silicon enhances rice resistance by reducing premature senescence and cell death. Additionally, high temperatures and drought conditions exacerbate the disease. With global warming and the increasing frequency of droughts, outbreaks of rice brown spot have become more frequent [3].

*Bipolaris oryzae* is a necrotrophic fungal pathogen capable of producing various virulence toxins, with ophiobolin A (OA) being one of its primary non-host-specific toxins [4]. Ophiobolin A exacerbates the development of rice brown spot by disrupting the structural integrity of host cell membranes, thereby creating a favorable environment for pathogen growth. In terms of host–pathogen interaction, *BMK1*, a gene encoding mitogen-activated protein kinase (MAPK), and its deletion in *B. oryzae* results in severe growth defects, including the loss of conidial production and reduced pathogenicity on rice leaves. These findings highlight the critical role of *BMK1* in sporulation and virulence in *B. oryzae* [5].

There has been significant progress in the study of resistance genes and quantitative trait loci (QTLs) associated with rice brown spot. These studies mainly involve hybrid strains of different rice varieties and disease-resistant cultivars, with QTL mapping conducted using molecular markers. For example, Mizobuchi et al. identified multiple resistance QTLs located on chromosomes 2, 9, and 11 of rice (such as qBS2, qBS9, and qBS11) through research on various rice varieties. These QTLs are closely associated with rice resistance to rice brown spot disease [6].

In plant pathogen research, the functional analysis of pathogen genomes is equally crucial. The genomic features of pathogens, including their core and accessory genomes, directly influence their ability to adapt to the environment and evade host immunity. With the rapid development of sequencing technologies, high-throughput sequencing (i.e., second-generation sequencing) has become one of the main tools for genomic research. However, the short-read lengths of second-generation sequencing limit the ability to analyze complex genomic structures, such as repetitive sequences and large segment rearrangements [7]. To overcome these limitations, third-generation sequencing technologies have emerged, including Single-Molecule Real-Time (SMRT) sequencing and Oxford Nanopore Technology (ONT). Compared to second-generation methods, the significant advantage of third-generation sequencing lies in its longer read lengths, providing unique benefits for de novo genome assembly and analysis of complex repetitive regions. This makes third-generation sequencing an indispensable tool for comprehensive analysis of complex genomes [8]. For example, by combining PacBio and Nanopore technologies, researchers achieved high-quality, telomere-to-telomere genome assembly of *Aspergillus fumigatus*, revealing complex chromosomal structures [9]. PacBio sequencing has also been used for the genome assembly of the rice pathogen *Magnaporthe oryzae*, exploring the potential role of transposable elements in genomic variation and virulence-related gene polymorphisms [10]. Similarly, using PacBio technology for sequencing *Pyricularia penniseti* strain P1609 helped identify key host-adaptation genes [11]. These studies demonstrate that third-generation sequencing technologies offer significant advantages in high-precision genome assembly and functional gene analysis of pathogenic fungi. Currently, genomic research on *Bipolaris oryzae* is limited, particularly the availability of high-quality genome data. Therefore, understanding the gene functions of *B. oryzae* is of great importance for elucidating its pathogenic mechanisms, adaptive variations, and interactions with rice.

Our study performed genome sequencing on the *Bipolaris oryzae* strain RBD1, collected from a dryland rice demonstration field in Haozhiba Village, Lancang County, Pu’er City, Yunnan Province. By constructing the complete genome of this strain and combining it with analyses of its pathogenicity and environmental adaptability under sorbitol osmotic stress, we further investigated the genetic features of the core genome of the rice sesame leaf spot pathogen. This study aims to lay the foundation for understanding the pathogenic mechanisms of *B. oryzae* and provide key scientific insights for the development of future disease control strategies.

## 2. Materials and Methods

### 2.1. Culture of Strain

The RBD1 strain, isolated from diseased rice leaves collected in a dryland rice demonstration field in Haozhiba Village, Lancang County, Pu’er City, Yunnan Province, was cultured on Potato Dextrose Agar (PDA) medium. The *Magnaporthe oryzae* H7 strain was obtained from our laboratory.

### 2.2. Genome Sequencing and Assembly

The purified RBD1 strain was inoculated onto PDA medium and incubated at 28 °C in a constant-temperature incubator for 5 days. Afterward, the mycelia were collected and transferred to Potato Dextrose Broth (PDB), then incubated at 28 °C while rotating at a speed of 120 r/min for 4 more days. The mycelia were collected again and washed 2–3 times with sterile water. Following the GP1 procedure, the genomic DNA was extracted using β-mercaptoethanol to disrupt cells and GP1 lysis buffer. Phenol/chloroform/isoamyl alcohol extraction and isopropanol precipitation were then performed. Centrifugation was used to separate the supernatant, the mixture was incubated at 65 °C, and additional purification procedures were performed. RNase A was used to remove RNA from the DNA after it had been precipitated with isopropanol at −80 °C and cleaned with ethanol. Qubit fluorometry and agarose gel electrophoresis were used for quality control, guaranteeing appropriate concentration and purity levels. Both the PacBio and Illumina platforms were utilized in the library development process. Using the SMRTbell™ Template Prep Kit 1.0 (100 reactions, Pacific Biosciences, Menlo Park, CA, USA), PacBio libraries were created by adapter ligation, DNA fragmentation, and damage repair. A minimum concentration of 1 ng/µL was required for the purified, size-selected, and quantified libraries prior to sequencing. The Illumina libraries were prepared using the NEBNext^®^ Ultra™ DNA Library Prep Kit (50 reactions, New England BioLabs, Ipswich, MA, USA)., which requires a minimum concentration of 2 ng/µL and involves comparable stages of fragmentation, end repair, adapter ligation, and PCR amplification. Qubit and Agilent 2100 systems (Agilent, Santa Clara, CA, USA) were used to verify insert size and quality. After passing quality control, the different libraries were sequenced using PacBio Sequel II/PacBio Sequel IIe and Illumina NovaSeq PE150 (Illumina, San Diego, CA, USA). Sequencing was carried out by Beijing Novogene Bioinformatics Technology Co., Ltd. (Beijing, China). For PacBio sequencing, a total of 3.5 Gb of long-read data were generated, corresponding to approximately 93.3× genome coverage (3.5 Gb/37.5 Mb). For Illumina sequencing, 11 Gb of paired-end reads were produced, providing approximately 293.3× genome coverage (11 Gb/37.5 Mb). These sequencing depths ensured sufficient data for accurate genome assembly and error correction. Low-quality reads were filtered using SMRT Link v8.0 software, and the filtered reads were assembled into contigs using Falcon v0.3.0 software

### 2.3. Gene Prediction

Gene component prediction, including the prediction of coding genes, repetitive sequences, and non-coding RNAs, was performed as follows: Augustus 2.7 software was used to predict coding genes; RepeatMasker (http://www.repeatmasker.org/, acceessd on 25 December 2023) was used to predict dispersed repetitive sequences; Tandem Repeats Finder (TRF) was used to analyze tandem repeats; tRNAscan-SE was used to predict transfer RNA (tRNA); rRNAmmer v1.2 was used to analyze ribosomal RNA (rRNA); and BLAST v2.14.0 was employed to align with the Rfam database to predict small RNAs, snRNA, and miRNA.

### 2.4. Gene Function Annotation

Gene functional annotation was performed using GO (Gene Ontology), KEGG (Kyoto Encyclopedia of Genes and Genomes), KOG (Clusters of Orthologous Groups), and TCDB (Transporter Classification Database). Whole-genome BLAST searches (E-value < 1 × 10^−5^, minimum alignment length > 40%) were used to align sequences against these databases. Secreted proteins were predicted using the SignalP database, while secondary metabolite gene clusters were identified using antiSMASH. For pathogenic fungi, pathogenicity was analyzed using the PHI (Pathogen–Host Interactions) database. KEGG and GO enrichment analyses of genes associated with the loss of pathogenicity were conducted using R (Version 4.2.2). Carbohydrate-active enzymes were predicted using the CAZy database.

### 2.5. Comparative Genomics Analysis

The genomes of *Botrytis cinerea* B05.10, *Verticillium dahliae* VdLs.17, *Pyricularia oryzae* 70-15, *Alternaria alternata* SRC1lrK2f, *Bipolaris sorokiniana* ND90Pr, *Bipolaris maydis* ATCC 48331, and *Bipolaris oryzae* ATCC 44560 were used in comparative genomic analysis. The criteria for selection were based on their relevance as major fungal pathogens, their diverse lifestyles (necrotrophic, hemibiotrophic, and biotrophic), and their ability to infect cereal crops. These genomes were retrieved from publicly available databases, and their inclusion helped to identify conserved and unique genomic features that contribute to pathogenicity and environmental adaptation. The phylogenetic tree was constructed using TreeBeST v1.9.2, with 1000 bootstrap replicates. Shared and unique genes were identified through rapid clustering analysis using CD-HIT, with a sequence similarity threshold of 50% for amino acid sequences and a length difference threshold of 0.7. A petal diagram was then generated to visualize the distribution of these genes. KEGG and GO enrichment analyses for shared and unique genes were conducted using R (Version 4.2.2).

### 2.6. Transcriptome Sample Preparation

Transcriptomic comparisons of *Magnaporthe oryzae* H7 and *Bipolaris oryzae* RBD1 were conducted due to their contrasting responses to environmental conditions. *Magnaporthe oryzae* thrives under high-temperature and high-humidity conditions, with minimal impact on drought-like scenarios. *Bipolaris oryzae* exhibits the opposite behavior, frequently occurring under dry conditions and low water activity. By comparing the transcriptional profiles of these two pathogens under stress conditions, we aimed to identify key genes and pathways that enable them to adapt to contrasting environments. This approach provides valuable insights into the molecular mechanisms underlying their pathogenicity and environmental resilience.

Activated strains H7 and RBD1 were inoculated onto PDA medium and cultured at 28 °C in a constant-temperature incubator for 5 days. Using a 5 mm punch, ten mycelial plugs were collected from each culture and transferred into a PDB medium for 3 days. Subsequently, five mycelial plugs were transferred to a PDB medium containing 1.2 mol/L sorbitol, while the remaining PDB cultures with the original mycelial plugs continued to be cultured. Both sets were incubated at 28 °C on a shaker at 120 rpm for 4 additional days. The mycelia were then collected, washed 2–3 times with sterile water, quickly frozen in liquid nitrogen, and stored at −80 °C for subsequent transcriptome sequencing analysis. Three biological replicates were performed for each sample group.

### 2.7. Transcriptome Sequencing and Differential Expression Analysis

For RNA extraction, we followed the protocol described by Wang et al. (2022), in which the RNA was extracted using RNeasy Plus minikit (Qiagen, Hilden, MA, USA) [12], following the manufacturer’s protocol for filamentous fungi. Briefly, about 100 mg of fungal mycelia were crushed in liquid nitrogen and lysed using Buffer RLC (Qiagen, Hilden, MA, USA), which is appropriate for tissues that need to solidify. To eliminate insoluble components, the lysate was centrifuged through a QIAshredder. RNA binding to RNeasy Mini spin columns was facilitated by the addition of ethanol, and impurities were removed by washing. Excellent RNA was eluted in RNase-free water, and Qubit fluorometry and agarose gel electrophoresis were used to evaluate the acceptable quality. At least 100 ng/μL of 28S and 18S rRNA bands had to be present and undamaged for RNA samples to be deemed acceptable. By adding dUTP to the second cDNA strand synthesis, strand-specific libraries were produced after mRNA was enriched using Oligo(dT) magnetic beads. A-tailing, adapter ligation, USER digestion, fragmentation, cDNA synthesis, end repair, and PCR amplification were all steps in the library preparation process. Up to 100 mg of fungal mycelia were crushed in liquid nitrogen and lysed using Buffer RLC, which is appropriate for tissues that need to solidify. To eliminate insoluble components, the lysate was centrifuged through a QIAshredder. RNA binding to RNeasy Mini spin columns was facilitated by the addition of ethanol, and impurities were removed by washing. Excellent RNA was eluted in RNase-free water, and Qubit fluorometry and agarose gel electrophoresis were used to evaluate the acceptable quality. At least 100 ng/μL of 28S and 18S rRNA bands had to be present and undamaged for RNA samples to be deemed acceptable. By adding dUTP to the second cDNA strand synthesis, strand-specific libraries were produced after mRNA was enriched using Oligo(dT) magnetic beads. A-tailing, adapter ligation, USER digestion, fragmentation, cDNA synthesis, end repair, and PCR amplification were all steps in the library preparation process. Following Illumina sequencing, raw sequencing data were generated from the imaging data using CASAVA for base calling, producing FASTQ files that contain both the sequence information and corresponding quality scores. The raw data may include reads with adapters, reads containing undetermined bases (N), and low-quality reads (where bases with a Qphred score ≤ 5 exceed 50%). Quality control of the raw data was performed using Trimmomatic to remove these low-quality reads. Subsequent analyses were conducted using the clean data, with quality metrics such as Q20, Q30, and GC content calculated.

The reference genome and gene annotation files were downloaded directly from public databases. HISAT2 (v2.0.5) was used to build the reference genome index, aligning the clean reads to the reference genome. HISAT2 integrates the gene annotation file to create a splice-aware alignment database, enhancing both efficiency and accuracy. Gene expression levels were quantified using StringTie (v1.3.3b), which employs a network flow algorithm to assemble transcripts and estimate transcript expression levels with high precision. Differential expression analysis was performed using the DESeq R package v1.46.0 [13]. DEGs with |log2FC| > 1 and false discovery rate (FDR) ≤ 0.05 were considered to be significantly different expressed genes. Gene Ontology (GO) and Kyoto Encyclopedia of Genes and Genomes pathway (KEGG) enrichment analyses were performed on the differentially expressed genes using ClusterProfiler R package v4.14.0 [14].

## 3. Results

### 3.1. Genome Assembly and Evaluation

At the contig assembly level, the final genome sequence of RBD1 had a total length of 37.5 Mbp, a G + C content of 49.39%, and consisted of 42 contigs, with a contig N50 of 2.0 Mb. Compared with the genome data of *Bipolaris oryzae* ATCC 44560 uploaded to JGI, the assembled RBD1 genome was longer, contained fewer contigs, and had a larger contig N50 (Table 1), indicating superior assembly quality compared to *Bipolaris oryzae* ATCC 44560.

We aligned the RBD1 reads to the assembled genome sequence and analyzed the GC content and read coverage depth. The results showed that the GC content was primarily distributed within the range of 40–60%, with no significant GC bias. Most regions had sequencing depths ranging from 50× to 100×, indicating that both the GC content and sequencing depth were within normal ranges (Figure S1). Genome assembly completeness was assessed using Benchmarking Universal Single-Copy Orthologs (BUSCOs), revealing 283 complete BUSCO genes and an overall genome completeness of 97.60%. Specifically, 283 were complete and single-copy BUSCOs, while 2 were fragmented and 5 were missing BUSCOs (Table S1).

### 3.2. Genome Component Prediction

**Figure 1:**
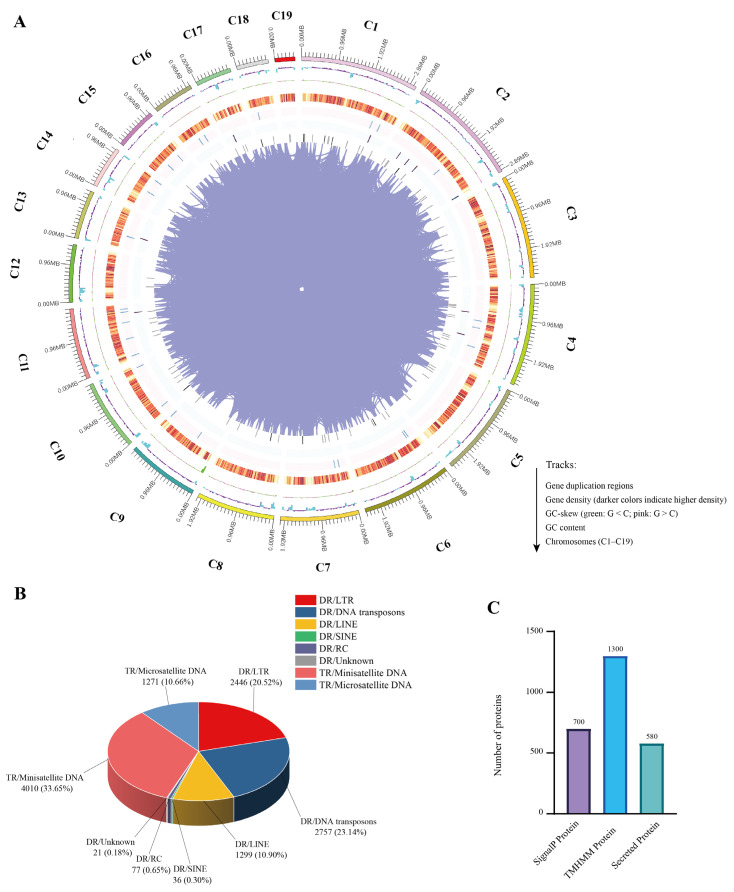
Statistics of genome component prediction. (**A**) Genomic map of RBD1. Circos (https://circos.ca/, accessed on 3 January 2024) was used for drawing. The outermost circle in the figure represents the 19 chromosomes of the genome (C1 to C19), arranged from largest to smallest based on genome size, with each small tick mark indicating 0.96 MB. The total genome size of RBD1 is shown to be 37.5 MB. The second circle displays the GC content, where light blue regions indicate GC content lower than the genome-wide average, and deep purple regions indicate higher GC content, with taller peaks signifying greater deviation from the average. The third circle illustrates the GC-skew, revealing the deviation between G and C content. Light green regions indicate areas where G content is lower than C content, while pink regions indicate the opposite. Moving inward, the next circles show gene density, including the distribution of coding genes, rRNA, snRNA, and tRNA, with darker colors indicating higher gene density. Finally, the innermost circle displays the gene duplication regions. (**B**) Statistical analysis and the percentage of different types of interspersed repeats in the RBD1. (**C**) Distribution of predicted proteins based on SignalP and TMHMM analyses. The x-axis represents categories of proteins: “SignalP Protein”, “TMHMM Structure”, and “Secreted Protein”. The “Secreted Protein” category includes proteins predicted to have signal peptides but no transmembrane domains. The y-axis indicates the number of proteins in each category.

We identified a total of 7605 coding genes with an average gene length of 1426 bp (Table S2) (Figure S2). Using the Circos v0.69-9 software, we visualized various genomic features of the RBD1 genome (Figure 1A). The outermost circle in the figure represents the 19 chromosomes of the genome (C1 to C19), arranged from largest to smallest based on genome size, with each small tick mark indicating 0.96 MB. The total genome size of RBD1 is shown to be 37.5 MB. The second circle displays the GC content, where light blue regions indicate GC content lower than the genome-wide average, and deep purple regions indicate higher GC content, with taller peaks signifying greater deviation from the average. The third circle illustrates the GC-skew, revealing the deviation between G and C content. Light green regions indicate areas where G content is lower than C content, while pink regions indicate the opposite, suggesting potential replication origin and termination sites. Moving inward, the next circles show gene density, including the distribution of coding genes, rRNA, snRNA, and tRNA, with darker colors indicating higher gene density. We also analyzed the distribution of repetitive sequences in the genome, predicting two major types: dispersed repeats (DRs) and tandem repeats (TRs). Dispersed repeats accounted for 55.69% of the genome, while tandem repeats constituted 44.31%. Among dispersed repeats, DNA transposons were the most abundant, comprising 23.14% of the genome. In tandem repeats, minisatellite DNA was the most prevalent, accounting for 33.65% of the genome (Figure 1B). These results provide a comprehensive overview of the structural and functional regions of the RBD1 genome, laying the foundation for further functional analyses and investigations into its pathogenicity and evolution.

In the functional annotation of the RBD1 genome, we performed an in-depth analysis of functional RNAs and secreted proteins to uncover their potential biological roles and contributions to pathogenicity. First, using rRNAmmer v1.2 software, we predicted rRNA regions within the genome, identifying three main types of rRNA: 5S rRNA, 18S rRNA, and 28S rRNA (Table 2). Among these, 5S rRNA was the most abundant, with a total of 29 copies identified. We identified 117 tRNA genes using tRNAscan-SE v2.0.12 software. Additionally, sRNA regions were predicted using Rfam v 12.1 software, which revealed only two sRNAs. To predict secreted proteins, we used SignalP (https://services.healthtech.dtu.dk/services/SignalP-6.0/, accessed on 10 September 2024) and TMHMM (https://services.healthtech.dtu.dk/services/TMHMM-2.0/, accessed on 10 September 2024)to detect the presence of signal peptides and transmembrane structures, respectively. The combined prediction identified proteins that contain signal peptides but lack transmembrane structures, suggesting they are secreted proteins (Figure 1C). Among these, 700 proteins contained signal peptides (Table S3), while 1330 proteins were predicted to have transmembrane structures (Table S4). Ultimately, 580 proteins were predicted as secreted proteins (Table S5). These secreted proteins may play crucial functional roles in the pathogenicity of plant pathogenic fungi, being secreted into the extracellular space to modulate host immune responses or degrade host cell wall components through secreted enzymes, thus facilitating infection [15,16].

### 3.3. Gene Functional Annotation

**Figure 2:**
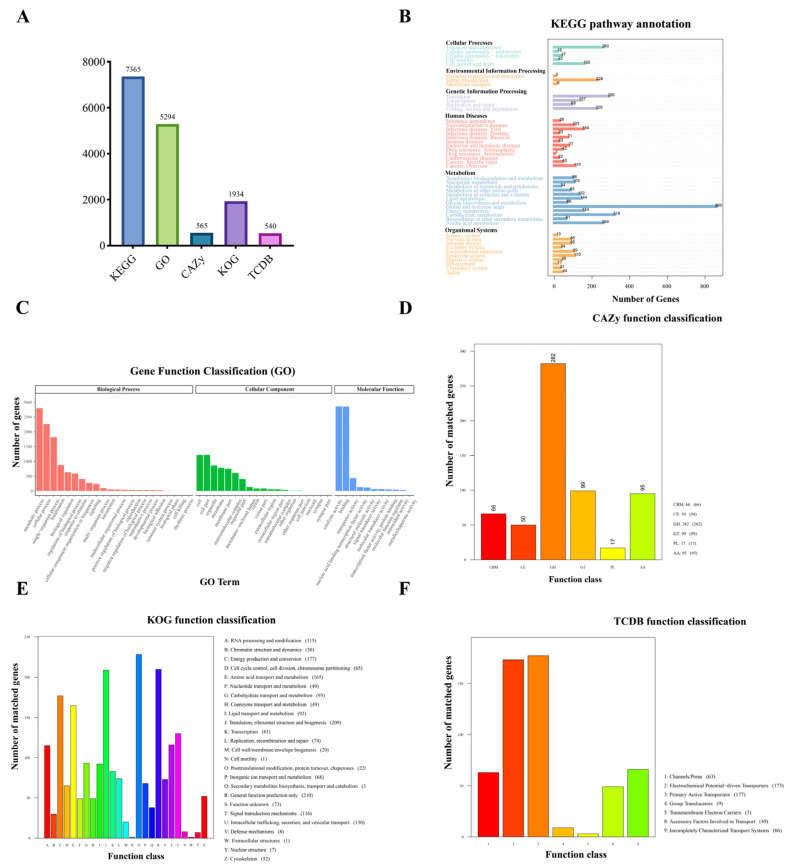
Annotation analysis of RBD1 in KEGG, GO, CAZy, KOG, and TCDB databases. (**A**) Number of genes annotated by RBD1 in various databases. (**B**) Gene function annotation of RBD1 by KEGG pathway. (**C**) Gene function annotation of RBD1 by GO annotation. (**D**) CAZy functional classification of RBD1. (**E**) Gene function annotation of RBD1 by KOG. (**F**) Gene function annotation of RBD1 by TCDB functional classification.

We performed multi-database annotation analyses of the RBD1 genome to comprehensively explore its potential biological processes and functional distribution characteristics (Figure 2A). In the Kyoto Encyclopedia of Genes and Genomes (KEGG) database (Figure 2B), a total of 7365 genes were annotated, with a significant enrichment in metabolism, particularly in the “Global and Overview Maps” category, which includes 860 genes. Among these, 319 genes are involved in carbohydrate metabolism, and 259 genes are associated with amino acid metabolism. Additionally, genes related to “Genetic Information Processing” were also highly enriched, especially in the processes of “Translation” and “Folding, Sorting, and Degradation”, with 290 and 226 genes, respectively. The Gene Ontology (GO) database annotation further supports the active roles of RBD1 in metabolism and cellular functions. A total of 5294 genes were annotated to different GO functional categories (Figure 2C). In terms of biological process, the genes were primarily enriched in metabolic processes, cellular processes, and single-organism processes. In the cellular component category, the genes were concentrated in “cell” and “cell part”, indicating their involvement in cellular structures and components. In the molecular function category, the genes were mainly enriched in catalytic activity and binding functions. In the carbohydrate-active enzymes (CAZy) database (Figure 2D), 565 genes in RBD1 were annotated as carbohydrate-active enzymes, with glycoside hydrolases (GHs) being the most dominant group, comprising 282 genes. These enzymes are likely to play an important role in the degradation of plant cell wall components, facilitating pathogen invasion and spread. Additionally, 99 genes were annotated as glycosyltransferases (GTs), and 95 genes were classified as auxiliary activity enzymes (AA). The Eukaryotic Orthologous Groups of proteins (KOG) database annotation results revealed potential features of RBD1 related to protein modification and structure (Figure 2E). A total of 1934 genes were annotated, with 228 genes involved in posttranslational modification, protein turnover, and chaperones. Additionally, 209 genes were annotated in the category of Translation, Ribosomal Structure, and Biogenesis. In the Transporter Classification Database (TCDB) (Figure 2F), a total of 489 genes related to transmembrane transport were annotated in RBD1, with the majority being Primary Active Transporters, comprising 177 genes.

In summary, the functional annotation results of the RBD1 genome indicate that its genes are primarily enriched in areas such as metabolic regulation, protein synthesis and modification, and intracellular and extracellular transport. The adaptability and pathogenic potential of RBD1 in complex ecosystems may be mediated by various biological processes and metabolic pathways.

### 3.4. Pathogenicity Analysis

**Figure 3:**
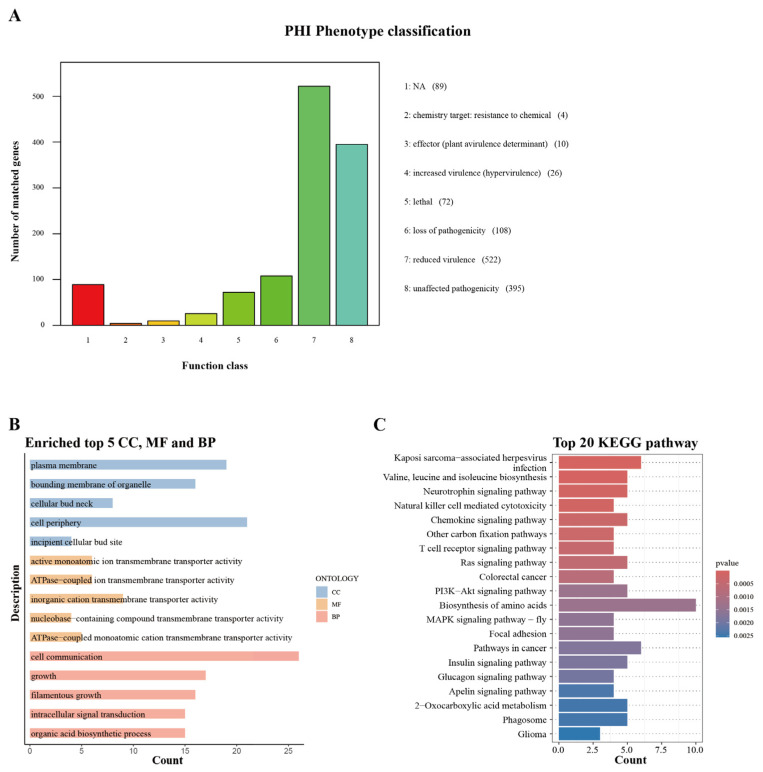
Pathogenicity analysis of RBD1 based on genome sequencing. (**A**) PHI pathotype classification of RBD1. (**B**) GO enrichment of 108 genes annotated as loss of pathogenicity in RBD1. (**C**) KEGG enrichment of 108 genes annotated as loss of pathogenicity in RBD1.

We used the PHI database to analyze the potential pathogenicity-related genes in RDB1 (Table S6). A total of 1226 genes annotated in the PHI database were classified based on their functions. Among these, 522 genes were annotated as “reduced virulence”, 395 genes as “unaffected pathogenicity”, 108 genes as “loss of pathogenicity”, and 89 genes were annotated as “unknown” (Figure 3A). A functional enrichment analysis (GO and KEGG) was conducted for the 108 genes annotated as contributing to the loss of pathogenicity. The GO enrichment analysis (Figure 3B) revealed that the “cellular component” category was significantly enriched in the plasma membrane, bounding membrane of organelles, cellular bud neck, cell periphery, and incipient cellular bud site. The “molecular function” (MF) category showed significant enrichment in activities such as active monoatomic ion transmembrane transporter activity, ATPase-coupled ion transmembrane transporter activity, inorganic cation transmembrane transporter activity, nucleobase-containing compound transmembrane transporter activity, and ATPase-coupled monoatomic cation transmembrane transporter activity. In the “biological process” category, the enrichment was mainly focused on processes such as cell communication, growth, filamentous growth, intracellular signal transduction, and organic acid biosynthesis. The KEGG enrichment analysis (Figure 3C) revealed that these genes were significantly enriched in various signaling pathways and metabolic pathways, including Kaposi sarcoma-associated herpesvirus infection, valine, leucine, and isoleucine biosynthesis, neurotrophin signaling pathway, natural killer cell mediated cytotoxicity, chemokine signaling pathway, other carbon fixation pathways, T cell receptor signaling pathway, Ras signaling pathway, and colorectal cancer. These enrichment results suggest that the genes responsible for the loss of pathogenicity in RDB1 are involved in processes such as transmembrane transport, cell communication, signal transduction, as well as multiple metabolic and signaling pathways.

Among the 108 genes related to the loss of pathogenicity, five genes exhibited >90% identity in RBD1 (Table 3). These include 3-isopropylmalate dehydrogenase, MAPK kinase kinase, G beta protein subunit, NADPH oxidase, and Tubulin alpha-1 chain.

### 3.5. Core Genes and Specific Genes Analysis

**Figure 4:**
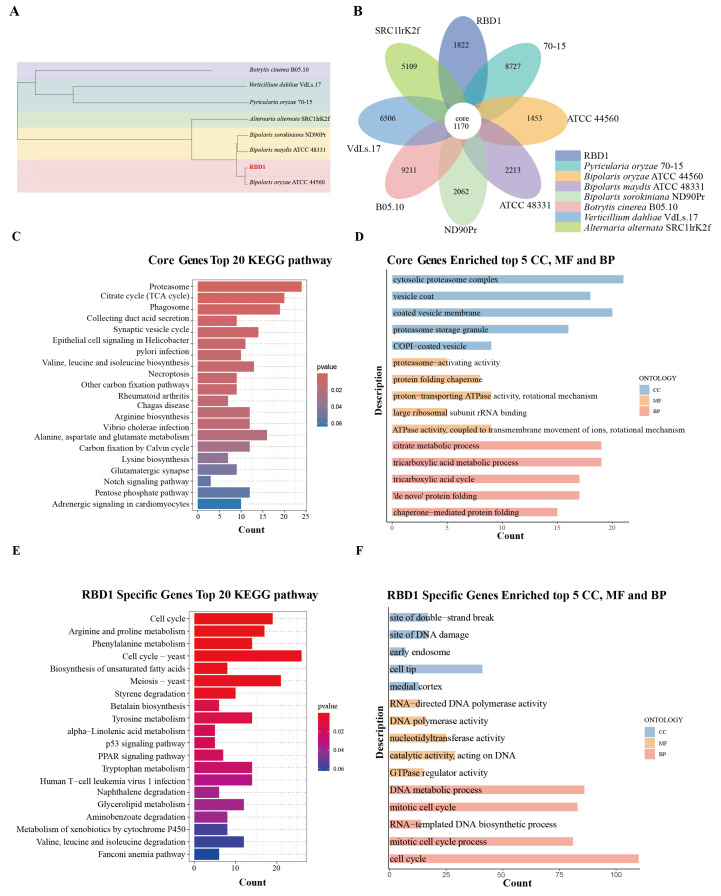
Core genes and specific genes based on comparative genome analysis. (**A**) Evolutionary relationship between *Pyricularia oryzae* 70-15, *Bipolaris oryzae* ATCC 44560, *Bipolaris maydis* ATCC 48331, *Bipolaris sorokiniana* ND90Pr, *Verticillium dahliae* VdLs.17, *Botrytis cinerea* B05.10, *Alternaria alternata* SRC1lrK2f, and RBD1. The phylogenetic tree was constructed using TreeBeST, with 1000 bootstrap replicates. (**B**) Core and specific genes of RBD1. (**C**) KEGG enrichment analysis of core genes. (**D**) GO enrichment analysis of core genes. (**E**) KEGG enrichment analysis of specific genes in RBD1. (**F**) GO enrichment analysis of specific genes in RBD1.

We used the genomes of seven fungal strains—*Pyricularia oryzae* 70-15, *Bipolaris oryzae* ATCC 44560, *Bipolaris maydis* ATCC 48331, *Bipolaris sorokiniana* ND90Pr, *Verticillium dahliae* VdLs.17, *Botrytis cinerea* B05.10, *Alternaria alternata* SRC1lrK2f, and RBD1—for analysis. A phylogenetic tree was constructed based on the single-copy core genes identified through corepan analysis (Figure 4A). The results showed that RBD1 clustered with *Bipolaris oryzae* ATCC 44560.

Upon analyzing the core and specific genes of the seven fungal strains and the target strain RBD1, the results indicated that there were 1170 core genes, while RBD1 contained 1822 specific genes (Figure 4B) (Tables S7 and S8). Functional annotation of the core genes revealed significant enrichment in several metabolic and signaling pathways in the KEGG database, including proteasome, citrate cycle (TCA cycle), phagosome, collecting duct acid secretion, synaptic vesicle cycle, epithelial cell signaling in helicobacter pylori infection, and valine, leucine and isoleucine biosynthesis (Figure 4C). For Gene Ontology (GO) annotation, cellular component (CC) enrichment was observed in structures such as the cytosolic proteasome complex and vesicle coat, while molecular function (MF) was primarily focused on proteasome-activating activity and protein-folding chaperone activities. Biological process (BP) enrichment included key metabolic processes such as citrate metabolic process, tricarboxylic acid cycle, and ‘de novo’ protein folding, suggesting the potential roles of the specific genes in fundamental metabolism and protein folding (Figure 4D).

In the analysis of RBD1′s specific genes, KEGG enrichment analysis showed that its specific genes were significantly enriched in pathways such as the cell cycle, arginine and proline metabolism, and phenylalanine metabolism, with particularly strong enrichment in the cell cycle yeast and biosynthesis of unsaturated fatty acids pathways. This suggests that RBD1 may adapt to its environment by regulating metabolism and the cell cycle (Figure 4E). Additionally, GO enrichment analysis revealed that the unique genes of RBD1 were enriched in biological processes (BPs) related to DNA metabolic process and mitotic cell cycle, while molecular functions (MFs) were concentrated on RNA-directed DNA polymerase activity and DNA polymerase activity. The cellular components (CCs) were mainly localized to the site of double-strand break and early endosome, suggesting that these specific genes may play important roles in DNA repair and intracellular membrane system functions (Figure 4F).

In contrast, specific genes from other strains displayed different enrichment patterns in KEGG and GO analyses. For instance, specific genes in *Pyricularia oryzae* 70-15 were enriched in KEGG pathways such as microRNAs in cancer and basal transcription factors, with GO annotations emphasizing active roles in RNA synthesis and transcription regulation. Specific genes in *Bipolaris oryzae* ATCC 44560 were primarily enriched in pathways related to drug metabolism and cell signaling, with GO analysis showing significant involvement in small nuclear ribonucleoprotein complex (snRNP) and intracellular transport proteins. Specific genes in *Alternaria alternata* SRC1lrK2f were enriched in functions related to exosome and metabolic regulation. In *Botrytis cinerea* B05.10, specific genes showed significant enrichment in pathways related to metabolic degradation and transcription regulation. Specific genes in *Verticillium dahliae* VdLs.17 were mainly enriched in KEGG pathways associated with cell cycle regulation, while GO annotations suggested potential specific functions in transcription regulation and chromatin structure maintenance. Specific genes in *Bipolaris sorokiniana* ND90Pr showed KEGG enrichment in pathways related to metabolic regulation and circadian rhythm control, and GO annotation suggested their involvement in nucleic acid metabolism and DNA synthesis (Figures S3–S8).

In conclusion, the specific genes of RBD1 are particularly involved in DNA repair and cell cycle regulation, which may confer a specific genetic advantage for its adaptation to specific habitats. On the other hand, other strains display varying strengths in pathways related to metabolic regulation, transcriptional control, and chromatin structure maintenance.

### 3.6. Differential Expression Analysis

**Figure 5:**
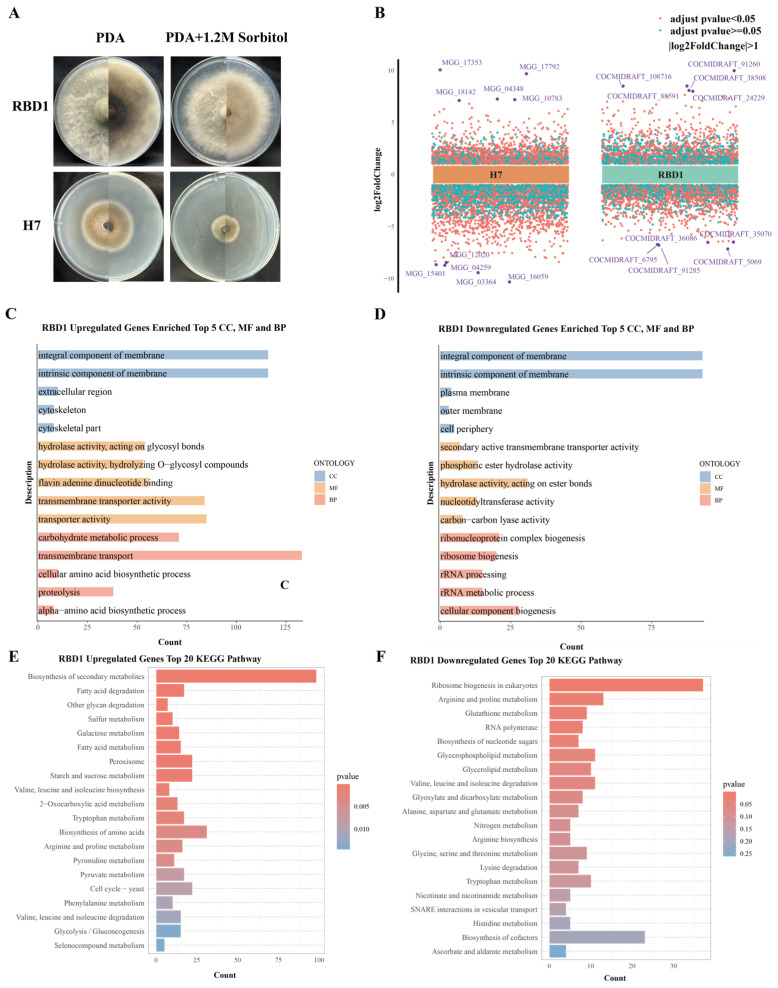
Differential expression analysis of RBD1 and H7 under 1.2 M sorbitol. (**A**) Phenotypes of RBD1 and H7 under 1.2 M sorbitol treatment. Measurements on day 6 of growth. (**B**) Multi-group volcano plot of differentially expressed genes of RBD1. Purple dots indicate the top 5 most significant upregulated and downregulated genes. (**C**,**D**) GO enrichment analysis of upregulated and downregulated genes in RBD1. (**E**,**F**) KEGG enrichment analysis of upregulated and downregulated genes in RBD1.

When 1.2 M sorbitol was added as a condition to simulate osmotic stress, the growth of *Magnaporthe oryzae* H7 was significantly inhibited, while *Bipolaris oryzae* RBD1 showed no significant inhibition (Figure 5A and Figure S9). To further investigate the response mechanisms of the two fungi under stress conditions, we performed transcriptome sequencing. After differential analysis, the results were visualized (Figure 5B). *Bipolaris oryzae* RBD1 had 1866 upregulated genes and 2201 downregulated genes (Tables S9 and S10), whereas *Magnaporthe oryzae* H7 had 2253 upregulated genes and 3198 downregulated genes (Tables S11 and S12). GO and KEGG enrichment analyses of the differentially expressed genes revealed the main biological functions and metabolic pathways involved in the differentially expressed genes of *Bipolaris oryzae* RBD1 and *Magnaporthe oryzae* H7 under sorbitol-induced osmotic stress (Figure 5C–F and Figure S10).

For RBD1, GO enrichment analysis of upregulated genes revealed significant enrichment in biological processes (BPs) related to carbohydrate metabolic process, transmembrane transport, cellular amino acid biosynthetic process, proteolysis, and alpha-amino acid biosynthetic process. Downregulated genes were primarily associated with processes such as ribonucleoprotein complex biogenesis, ribosome biogenesis, and rRNA processing. In terms of cellular components (CCs), upregulated genes were concentrated in the integral and intrinsic component of membrane, cytoskeleton and cytoskeletal part, and extracellular region, while downregulated genes were enriched in the outer membrane, plasma membrane, and cell periphery. Molecular function (MF) analysis showed that upregulated genes were significantly related to hydrolase activity and transmembrane transporter activity, while downregulated genes were concentrated in secondary active transmembrane transporter activity and nucleotidyltransferase activity. KEGG pathway analysis further revealed that upregulated genes were mainly involved in the biosynthesis of secondary metabolites, fatty acid degradation, and other glycan degradation, while downregulated genes were associated with ribosome biogenesis in eukaryotes, arginine and proline metabolism, and glutathione metabolism. For H7, GO analysis of upregulated genes indicated significant enrichment in biological processes (BPs) such as peptide metabolic process, translation, peptide biosynthetic process, amide biosynthetic process, and cellular amide metabolic process. In terms of cellular components (CCs), upregulated genes were significantly enriched in the ribosome, cytoplasmic part, and protein-containing complex. MF analysis revealed that upregulated genes were notably associated with structural constituent of ribosome, structural molecule activity, and proton transmembrane transporter activity. In contrast, downregulated genes were mainly enriched in the integral and intrinsic component of membrane and plasma membrane at the cellular component level. In biological processes (BPs), they were concentrated in transmembrane transport and regulation of metabolic and nitrogen compound metabolic processes. MF analysis showed that downregulated genes were closely related to transition metal ion binding, heme binding, and tetrapyrrole binding. KEGG pathway analysis further indicated that upregulated genes in H7 were primarily involved in ribosome, oxidative phosphorylation, and citrate cycle (TCA cycle), while downregulated genes were enriched in ABC transporters, biosynthesis of various plant secondary metabolites, and starch and sucrose metabolism.

These results suggest that RBD1 may effectively adapt to osmotic pressure changes and nutrient resource limitation under sorbitol-induced osmotic stress by enhancing metabolic functions (e.g., carbohydrate metabolism and fatty acid degradation), stabilizing cellular structures (e.g., adjusting components of the cell membrane and extracellular region), and activating substance degradation and transport. In contrast, H7 attempts to cope with the stress by enhancing protein synthesis (e.g., ribosome function and translation) and optimizing metabolic regulation.

### 3.7. Protein Interaction Network Analysis

**Figure 6:**
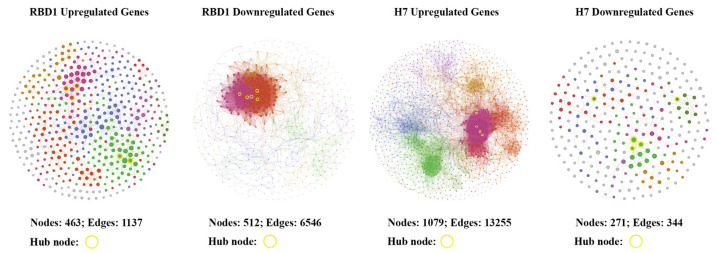
Protein network interaction diagram of upregulated and downregulated genes of RBD1 and H7.

To investigate the protein–protein interaction (PPI) network of *Magnaporthe oryzae* H7 and *Bipolaris maydis* RBD1 under sorbitol-induced osmotic stress, we constructed the corresponding PPI networks using differentially expressed genes (DEGs) and performed visualization analysis using Gephi (0.1.0) software (Figure 6) (Table 4). In terms of network topology, the upregulated gene network of H7 had a higher number of nodes (1079) and edges (13,255) compared to RBD1 (463 nodes, 1137 edges). This indicates that *Magnaporthe oryzae* H7 has a more complex network structure when responding to osmotic stress, involving more redundant pathways. In contrast, the upregulated gene network of RBD1 was more streamlined, suggesting a more simplified response mechanism.

Modularity is a key parameter that reflects the degree to which a network is divided into tightly connected modules or communities. A higher modularity value in the downregulated gene network of H7 (0.89) suggests that its response to osmotic stress is compartmentalized into specific functional modules. In contrast, the downregulated gene network of RBD1 has a lower modularity (0.21), indicating fewer distinct modules.

The average clustering coefficient, which measures the likelihood of nodes clustering together, is higher in the downregulated gene network of RBD1 (0.59) compared to H7 (0.49). This suggests that RBD1′s network maintains tighter local connections. On the other hand, H7 exhibits a more distributed network topology.

These topological differences illustrate distinct strategies employed by H7 and RBD1 under osmotic stress. H7′s network topology indicates more complex, modular response, while RBD1′s network emphasizes tightly connected pathways.

## 4. Discussion

In the annotation of the genome of *Bipolaris oryzae* RBD1, we predicted a total of 7605 coding genes. These genes are involved in a wide range of functions, including metabolism, signal transduction, gene regulation, and cellular structure, providing a crucial foundation for understanding the complex biological characteristics of this pathogenic fungus. Specifically, many of the functional genes are associated with enzymes involved in basic metabolic processes, such as carbon, nitrogen, and phosphorus metabolism, as well as genes involved in lipid metabolism and secondary metabolic pathways. This suggests that *Bipolaris oryzae* exhibits metabolic flexibility and adaptability to its host plants. Additionally, several genes related to signal transduction and gene expression regulation were identified, including those encoding transcription factors, kinases, and phosphatases. These genes may play key roles in regulating the growth, environmental adaptability, and pathogenicity of *Bipolaris oryzae* RBD1. It is important to note that while these coding genes were predicted using established genome annotation tools, experimental validation to confirm their ability to produce functional proteins was not conducted in this study. This limitation highlights the need for future research using experimental approaches, such as protein expression assays, to validate the functional predictions made here.

In this study, our in-depth analysis of the pathogenicity-related genes of *Bipolaris oryzae* RBD1 revealed genes closely associated with its pathogenicity and their functional characteristics. By screening the predicted pathogenicity-related genes, we identified 108 genes annotated as genes responsible for loss of pathogenicity in other pathogenic fungi, with 5 genes showing sequence similarity (identity) greater than 90%. These genes are involved in various important functions related to the pathogenic process, such as the synthesis of secondary metabolites, regulation of signal transduction pathways, redox reactions, and maintenance of cellular structures. Specifically, the functions of these high-similarity genes include 3-isopropylmalate dehydrogenase, an enzyme crucial for leucine biosynthesis [17]. Leucine, an essential component of proteins, also plays a role in regulating fungal cell responses to environmental stress [18]. Studies have shown that changes in leucine levels can affect plant responses to environmental stress, such as the dynamic changes in leucine content observed in ABA-resistant barley grains, which are associated with stress responses [19]. These findings support the notion that the leucine pathway in RBD1 may contribute to environmental stress adaptation and potentially assist RBD1 in maintaining its pathogenicity and survival advantage under osmotic stress conditions in rice. MAPK kinase is another gene identified in this study. The MAPK signaling pathway plays a critical role in the interaction between pathogenic fungi and their host plants. In *Magnaporthe oryzae*, studies have shown that mutant strains lacking the MEK *mst11* and MAPK *mst7* genes cannot form appressoria and completely lose pathogenicity [20]. Similarly, in *Puccinia striiformis f.* sp. *tritici*, PsKPP4, a STE11-homologous MAPKKK regulates infection-related morphogenesis and pathogenicity through interaction with PsUBC2 [21]. Furthermore, the MAPK signaling cascade can activate downstream transcription factors and effectors to initiate gene expression programs that allow adaptation to environmental changes [22]. The presence of this highly conserved signaling mechanism in RBD1 may indicate that this strain has the ability to rapidly respond to host plant resistance mechanisms or environmental stresses, thereby enhancing its pathogenicity or survival adaptability. The G beta protein subunit is another important gene involved in the G protein signaling pathway, which is crucial for the perception of external environmental changes and regulation of developmental and pathogenic processes in pathogenic fungi [23]. As a key component of this signaling pathway, the G beta subunit participates in the fungal perception of host plant chemical signals and plays an important role in regulating infection structure formation and toxin secretion. For instance, in *Botrytis cinerea*, Gβ-like proteins (e.g., Bcgbl1) affect development and pathogenicity by regulating the MAP kinase signaling pathway [24]. In *Aspergillus fumigatus*, the complex formed by the Gβ and Gγ subunits regulates spore formation, toxin production (e.g., gliotoxin), and stress responses, which are critical for pathogenic fungal infections [25]. NADPH oxidase, an enzyme crucial for the generation of reactive oxygen species (ROS), plays an important role in the interaction between pathogens and host plants. Research indicates that NADPH oxidase, as the main enzyme responsible for ROS production, influences the synthesis of cell wall-degrading enzymes by regulating ROS signaling, thereby enhancing the pathogenicity of the pathogen [26]. Additionally, ROS production and regulation play a significant role in the pathogen’s ability to counteract the host’s antioxidant defense, maintaining the oxidative balance between the pathogen and the host plant. This interplay not only influences the pathogenic process but also determines whether the pathogen can successfully infect the host [27]. Tubulin, a key component of the microtubule cytoskeleton, is essential for maintaining cell shape, cell division, and intracellular transport [28]. The protein-α-tubulin plays an indispensable role in fungal growth, conidiation, and the formation of infection structures, such as appressoria and invasive hyphae [29]. Its stability may be closely related to RBD1′s ability to colonize host plants. These findings of pathogenicity-related genes not only provide a molecular basis for understanding the pathogenic mechanisms of *Bipolaris oryzae*, but also offer direction for further studies on key regulatory nodes in the fungal–plant interaction process. Furthermore, to gain a more comprehensive understanding of the functions of these genes, we conducted Gene Ontology (GO) and KEGG pathway enrichment analyses. The results revealed significant enrichment of pathogenicity-related genes in GO terms closely associated with infection, cell wall degradation, toxin synthesis, and signal transduction. This suggests that these genes play an important role in the fungal infection process, particularly in regulating the formation of fungal infection structures and the degradation of host plant cell walls. KEGG pathway enrichment analysis further indicated significant enrichment in secondary metabolic pathways (e.g., polyphenol synthesis, lipid metabolism), MAPK signaling pathways, and G protein-mediated signal transduction pathways. Secondary metabolic pathways are closely related to fungal toxin synthesis [30], while the MAPK signaling pathway plays an important role in regulating the fungal response to host defense mechanisms [31].

The enrichment of *Bipolaris oryzae* RBD1-specific genes in amino acid metabolism and cell cycle-related pathways may reflect its adaptive capacity to environmental changes. These genes could help RBD1 maintain its survival and pathogenicity under fluctuating nutritional conditions or increased environmental stress by regulating intracellular metabolic balance and proliferation rates. For instance, the high expression of genes related to amino acid metabolism may enable RBD1 to rapidly adapt to nitrogen-limited environments within host tissues, a mechanism also observed in other pathogenic fungi [32]. Furthermore, cell cycle regulation is closely linked to the pathogenic process. In the maize anthracnose fungus *Ustilago maydis*, the cell cycle regulator Cln1 not only participates in regulating cell growth but also coordinates the relationship between cell cycle progression and pathogenic development. This mechanism helps the fungus make adaptive developmental choices based on varying environmental conditions [33]. A similar regulatory strategy may also be present in RBD1, enabling it to respond more flexibly and maintain pathogenicity in complex environments. These findings not only contribute to understanding the pathogenic mechanisms of *Bipolaris oryzae*, but also provide potential target genes for the development of future disease control strategies.

Fungal adaptation to osmotic stress has been extensively studied in extremophilic and osmotolerant fungi, such as *Aspergillus sydowii* and *Wallemia ichthyophaga*, which thrive in environments with extreme salinity or low water activity. These fungi are valuable models for understanding how fungi respond to osmotic stress. For example, under high salinity, *A. sydowii* accumulates osmolytes such as glycerol, trehalose, and mannitol to maintain osmotic balance [34]. Additionally, its antioxidant defense system is activated, with enzymes like catalase and superoxide dismutase upregulated to mitigate oxidative stress induced by salinity [35,36]. Similarly, the HOG signaling pathway in *W. ichthyophaga* plays a crucial role in osmoadaptation under salt stress, while structural changes in its cell wall improve cellular integrity in high-salinity environments [37]. In *Bipolaris oryzae* RBD1, genes involved in osmotic stress adaptation are significantly upregulated, including those in the “arginine and proline metabolism” pathway, which is critical for proline synthesis, and the “starch and sucrose metabolism” pathway, which may contribute to trehalose biosynthesis from sucrose or other sugar derivatives. The “peroxisome” pathway, involved in redox metabolism, likely supports antioxidant defenses, including reactive oxygen species (ROS) scavenging. Additionally, the “2-Oxocarboxylic acid metabolism” pathway, linked to redox reactions, may help mitigate oxidative stress. These findings underscore the upregulation of genes involved in osmolyte biosynthesis (e.g., proline and trehalose), antioxidant defenses (e.g., glutathione and thioredoxin systems), and metabolic pathways that sustain energy production and cellular integrity. Although this study provides insights into the transcriptional responses of *B. oryzae* under osmotic stress, several areas warrant further investigation. For instance, while key pathways such as “arginine and proline metabolism” and “starch and sucrose metabolism” were identified, their specific roles in fungal adaptation to low water activity still need to be clarified. The contribution of these pathways to osmolyte production and osmotic balance, particularly under conditions that mimic natural environments, requires more targeted experimental validation.

Although the transcriptomic analysis identified differentially expressed genes (DEGs) under osmotic stress, no experimental validation, such as RT-qPCR, was conducted in this study. This represents a limitation of the current work. Future studies should validate the key DEGs using experimental approaches to strengthen the reliability of the findings and provide deeper insights into the molecular mechanisms underlying osmotic stress response in *Bipolaris oryzae*.

Overall, through the genome and transcriptome sequencing of *Bipolaris oryzae* RBD1, we identified genes related to carbon, nitrogen, and lipid metabolism, indicating that RBD1 possesses strong metabolic flexibility to adapt to different environmental conditions and host plants. Furthermore, transcriptome analysis revealed genes associated with signal transduction, cell wall degradation, and secondary metabolite synthesis, whose roles in the infection process warrant further investigation. This study also identified several genes closely linked to pathogenicity, such as those involved in the MAPK signaling pathway, G protein signaling pathway, and oxidative stress response, providing molecular evidence for understanding the interaction between RBD1 and rice, as well as its adaptive mechanisms. These findings not only deepen our understanding of the pathogenic mechanisms of *Bipolaris oryzae*, but also lay the foundation for future studies on its function and the development of more effective disease control strategies. However, there are still limitations in data interpretation and functional validation. Future research should further explore the specific roles of these genes in pathogenicity.

## Figures and Tables

**Table 1:** Genome assembly comparisons of two strains, RBD1 and ATCC 44560.

Features	RBD1	ATCC 44560
Contigs	42	671
Contig N50 Length	2.0 Mb	131.7 kb
GC (%)	49.39%	50.5%
Assembly level	Contig	Scaffold
Total length (Mbp)	37.5	31.4

**Table 2:** Predicted ncRNA in RBD1.

Type of ncRNA	Number	Average Length (bp)	Total Length (bp)
tRNA	117	88	10,333
sRNA	2	245	491
snRNA	11	153	1686
5s (de novo)	29	116	3365
28s (de novo)	3	4687	14,060
18s (de novo)	2	1806	3612

**Table 3:** Annotation on top 5 genes with loss of pathogenicity classified by PHI database.

Gene_Id	Gene Name	Pathogen Species	Host Species	Gene Function
A2992	IPMDH	*Parastagonospora nodorum*	*Triticum* (related: wheat)	3-isopropylmalate dehydrogenase
A4584	Csste11	*Bipolaris sorokiniana*	*Hordeum vulgare* (related: barley)	MAPK kinase kinase
A6894	CGB1	*Bipolaris maydis*	*Zea mays* (related: maize)	G beta protein subunit
A1398	NoxA	*Alternaria alternata*	*Pyrus communis* (related: pear)	NADPH oxidase
A3209	TUB1	*Aspergillus fumigatus*	*Mus musculus* (related: house mouse)	Tubulin alpha-1 chain

**Table 4:** Interaction network analysis for H7 and RBD1.

Topology Properties	H7		RBD1	
	Upregulated Genes	Downregulated Genes	Upregulated Genes	Downregulated Genes
Number of edges	13,255	344	1137	6546
Number of nodes	1079	271	463	512
Average Degree	24.57	2.54	4.91	25.57
Average Weighted Degree	24.57	2.54	4.91	25.57
Network Diameter	12	11	15	14
Graph density	0.02	0.01	0.01	0.05
Connected Components	18	44	26	24
Modularity	0.49	0.89	0.78	0.21
Statistical Inference	40,768.73	2024.02	5548.30	16,043.07
Average Clustering Coefficient	0.57	0.49	0.50	0.59
Average path length	3.99	3.83	6.15	4.63

## Data Availability

The raw data of genome and transcriptome sequencing are uploaded and they will be published when the manuscript is accepted.

## Supplementary Materials

The following supporting information can be downloaded at https://www.mdpi.com/article/10.3390/jof11030227/s1, Figure S1: GC content and read coverage depth of RBD1; Figure S2: Gene length distribution of RBD1; Figure S3: KEGG and GO enrichment of specific genes in *Pyricularia oryzae* 70-15; Figure S4: KEGG and GO enrichment of specific genes in *Bipolaris oryzae* ATCC 44560; Figure S5: KEGG and GO enrichment of specific genes in *Alternaria alternata* SRC1lrK2f; Figure S6: KEGG and GO enrichment of specific genes in *Botrytis cinerea* B05.10; Figure S7: KEGG and GO enrichment of specific genes in *Verticillium dahliae* VdLs.17; Figure S8: KEGG and GO enrichment of specific genes in *Bipolaris sorokiniana* ND90Pr; Figure S9: Colony diameter analysis of H7 and RBD1 under different growth conditions; Figure S10: GO and KEGG enrichment analyses of the up-regulated and down-regulated genes in H7 treated with 1.2 M sorbitol. Table S1: BUSCO statistics; Table S2: Predicted coding genes in RBD1; Table S3: Genes identified as signal peptide structural proteins of RBD1; Table S4: Genes identified as proteins with transmembrane structures of RBD1; Table S5: Genes predicted to be secreted proteins of RBD1; Table S6: PHI database annotation of RBD1; Table S7: Core gene; Table S8: Specific gene; Table S9: Upregulated genes of *Bipolaris oryzae* RBD1 in 1.2 M sorbitol treatment; Table S10: Downregulated genes of *Bipolaris oryzae* RBD1 in 1.2 M sorbitol treatment; Table S11: Upregulated genes of *Magnaporthe oryzae* H7 treated with 1.2 M sorbitol; Table S12: Downregulated genes of *Magnaporthe oryzae* H7 treated with 1.2 M sorbitol.
