# Supplementary figures and images for: Genome Sequencing and Comparative Transcriptomic Analysis of Rice Brown Spot Pathogen Bipolaris oryzae Adaptation to Osmotic Stress
Source: J Fungi (Basel). 2025 Mar 17;11(3):227. doi: 10.3390/jof11030227 (PMC11943334; doi:10.3390/jof11030227)

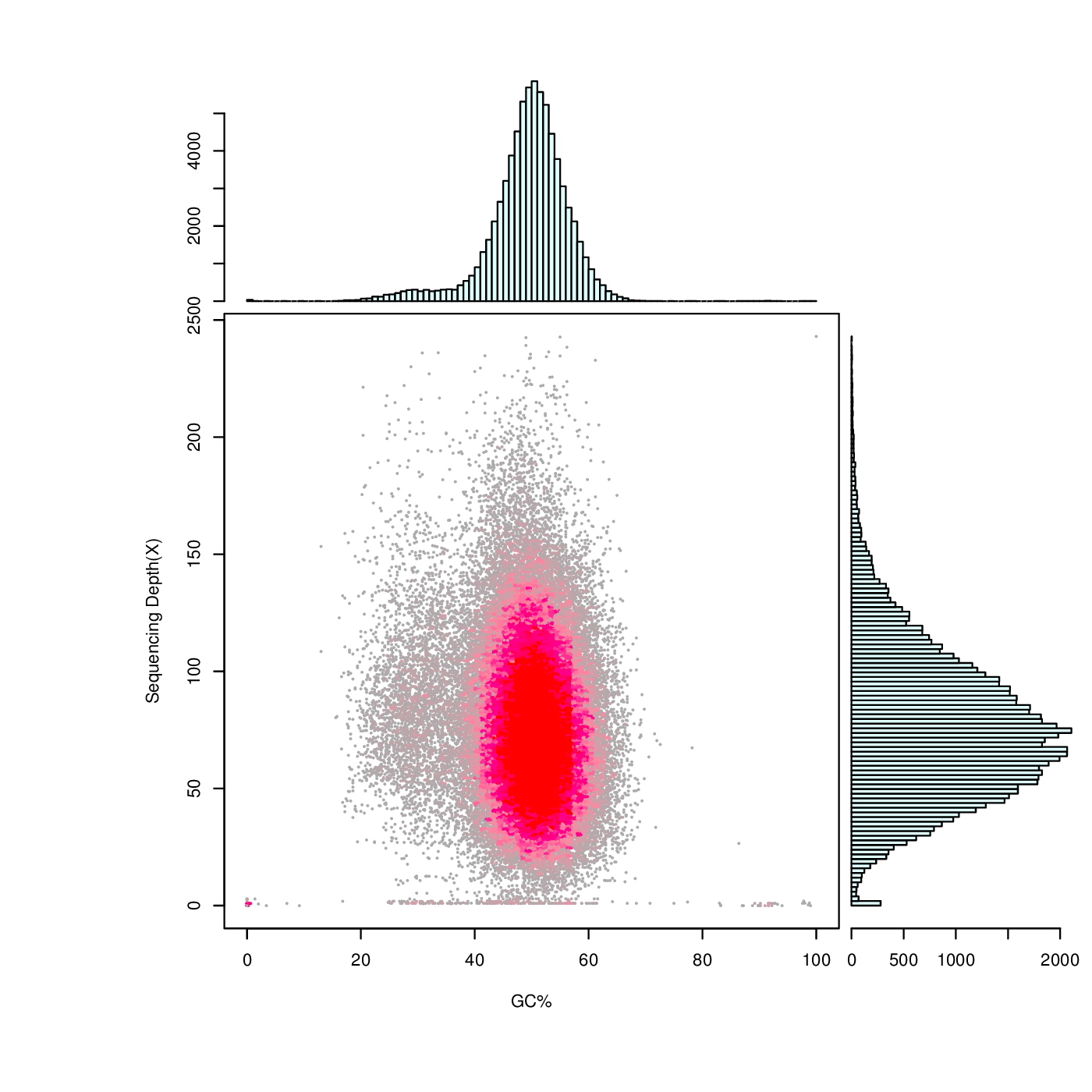

Supplement: Supplementary file 1 [file jof-11-00227-s001.zip › Supplementary/Figure S1.png]

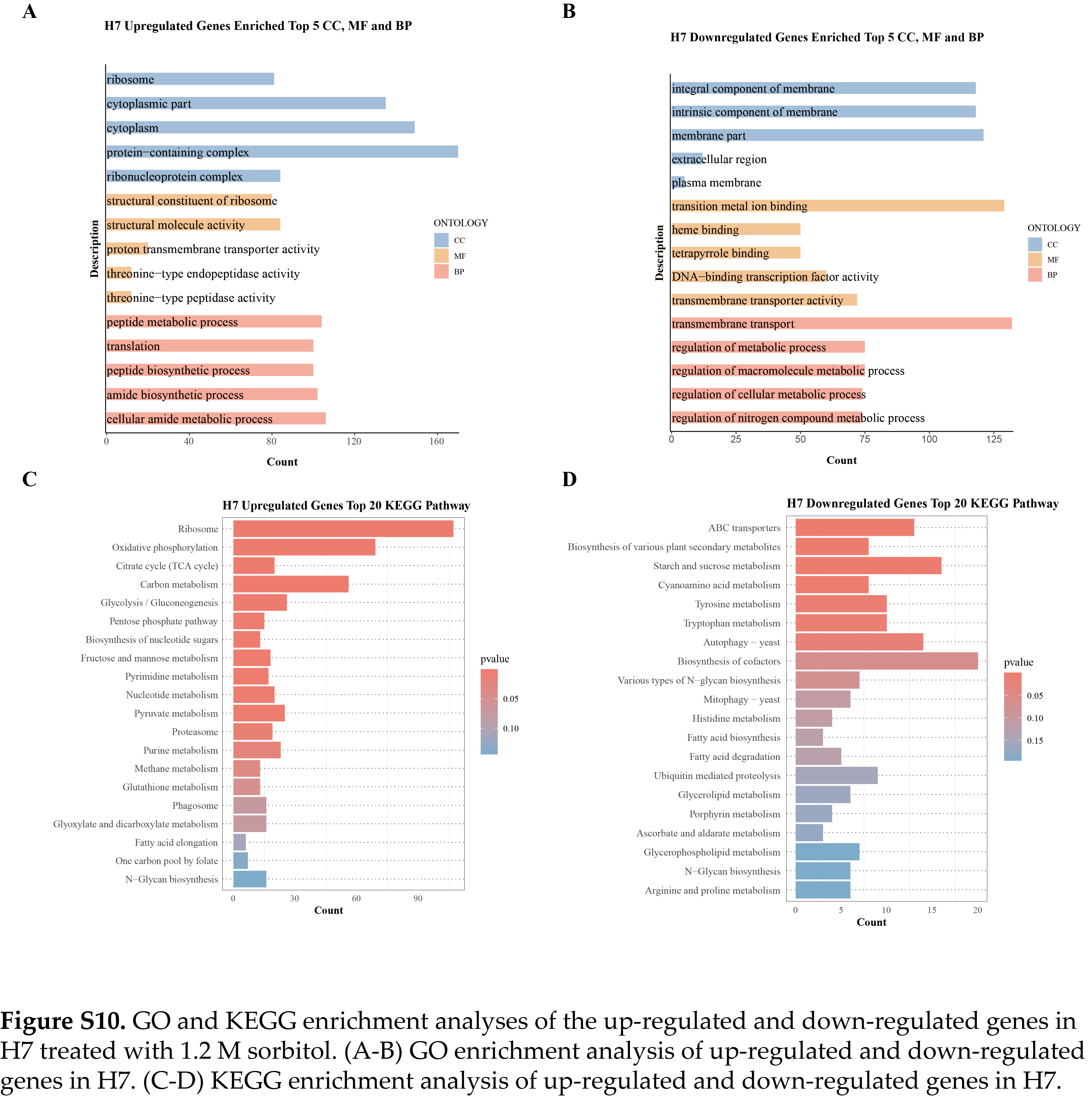

Supplement: Supplementary file 1 [file jof-11-00227-s001.zip › Supplementary/Figure S10.png]

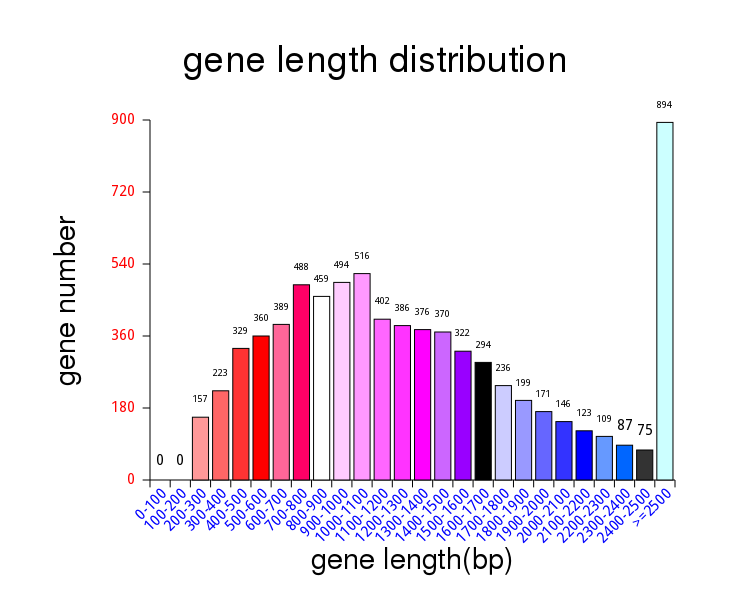

Supplement: Supplementary file 1 [file jof-11-00227-s001.zip › Supplementary/Figure S2.png]

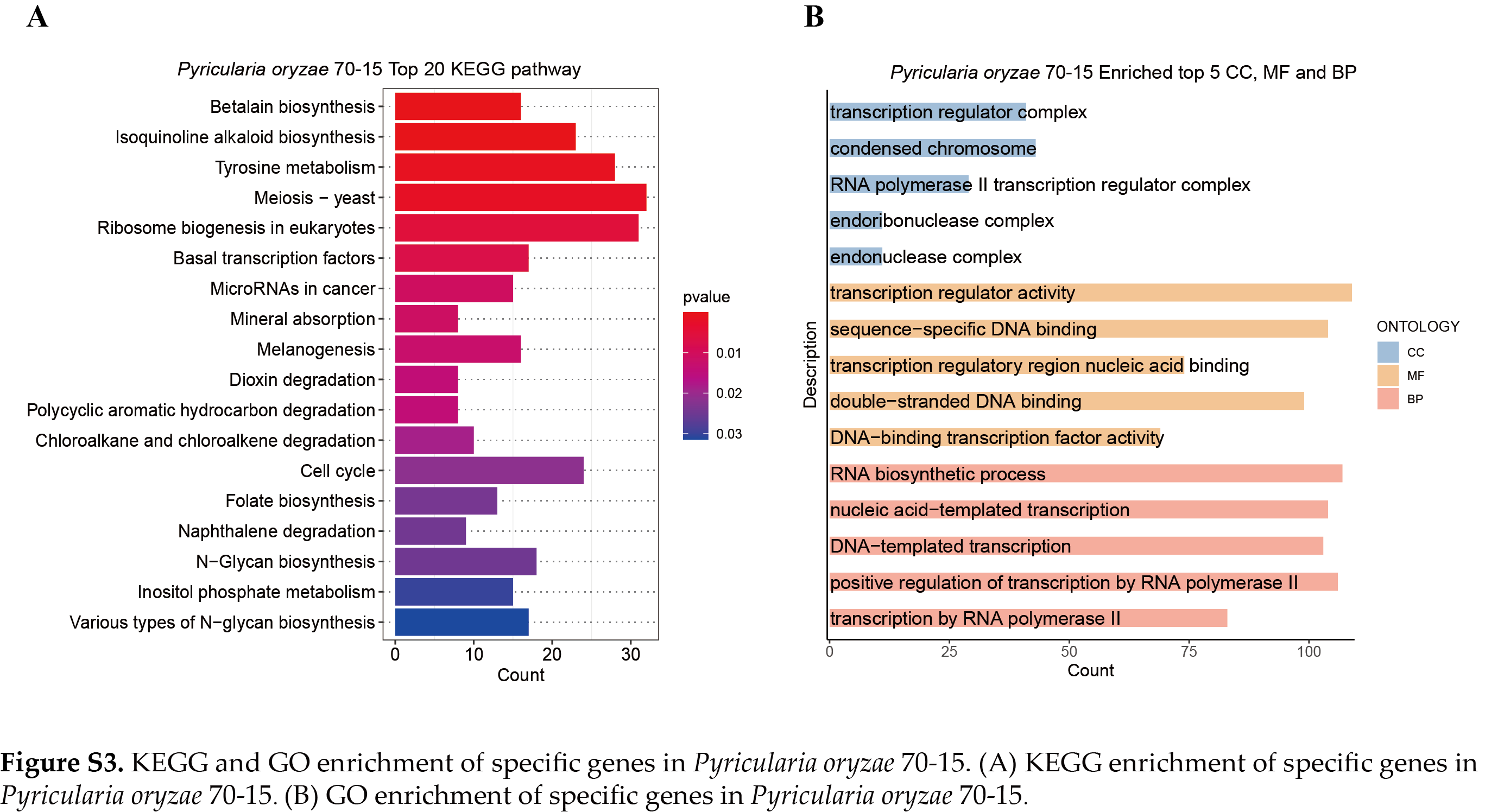

Supplement: Supplementary file 1 [file jof-11-00227-s001.zip › Supplementary/Figure S3.png]

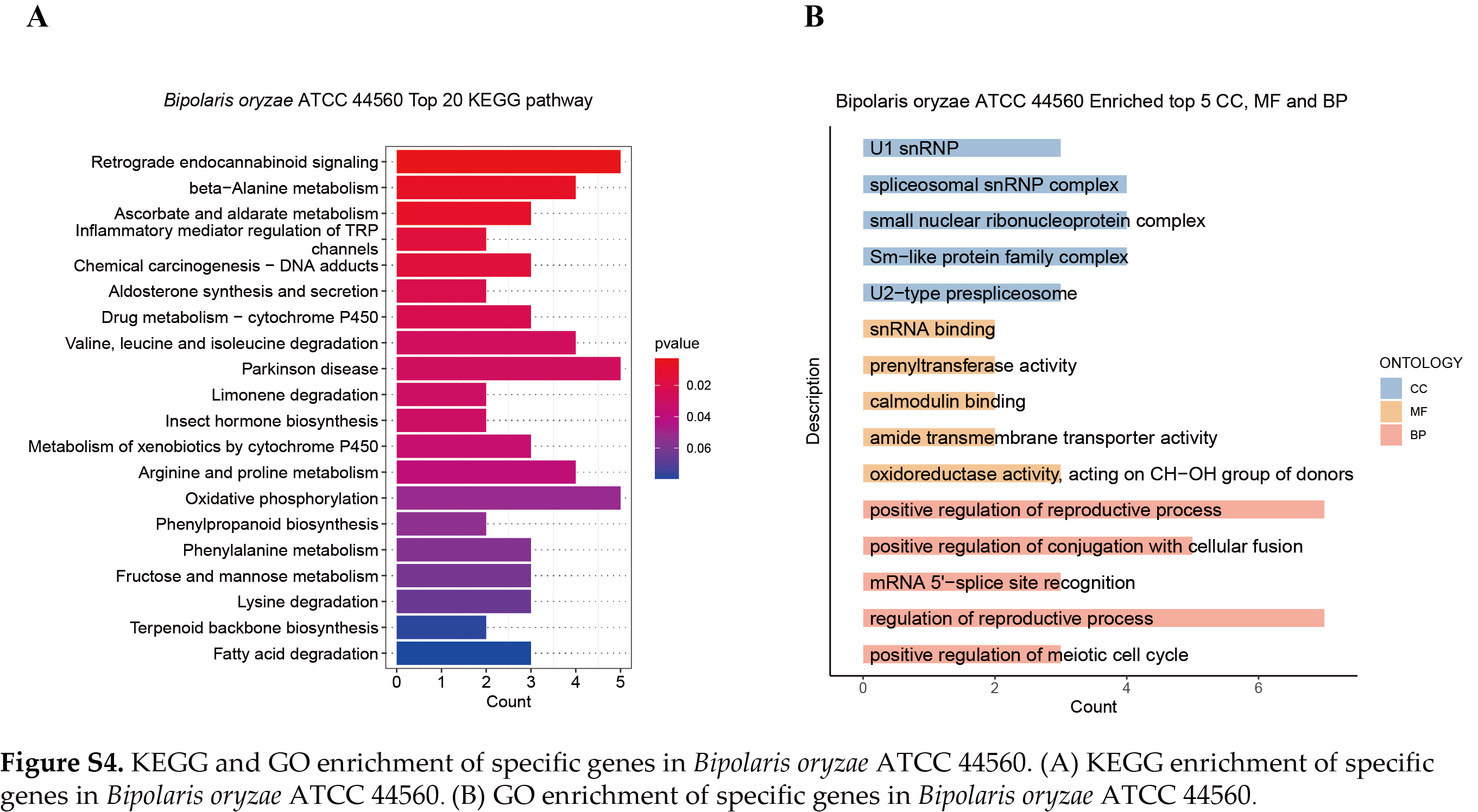

Supplement: Supplementary file 1 [file jof-11-00227-s001.zip › Supplementary/Figure S4.png]

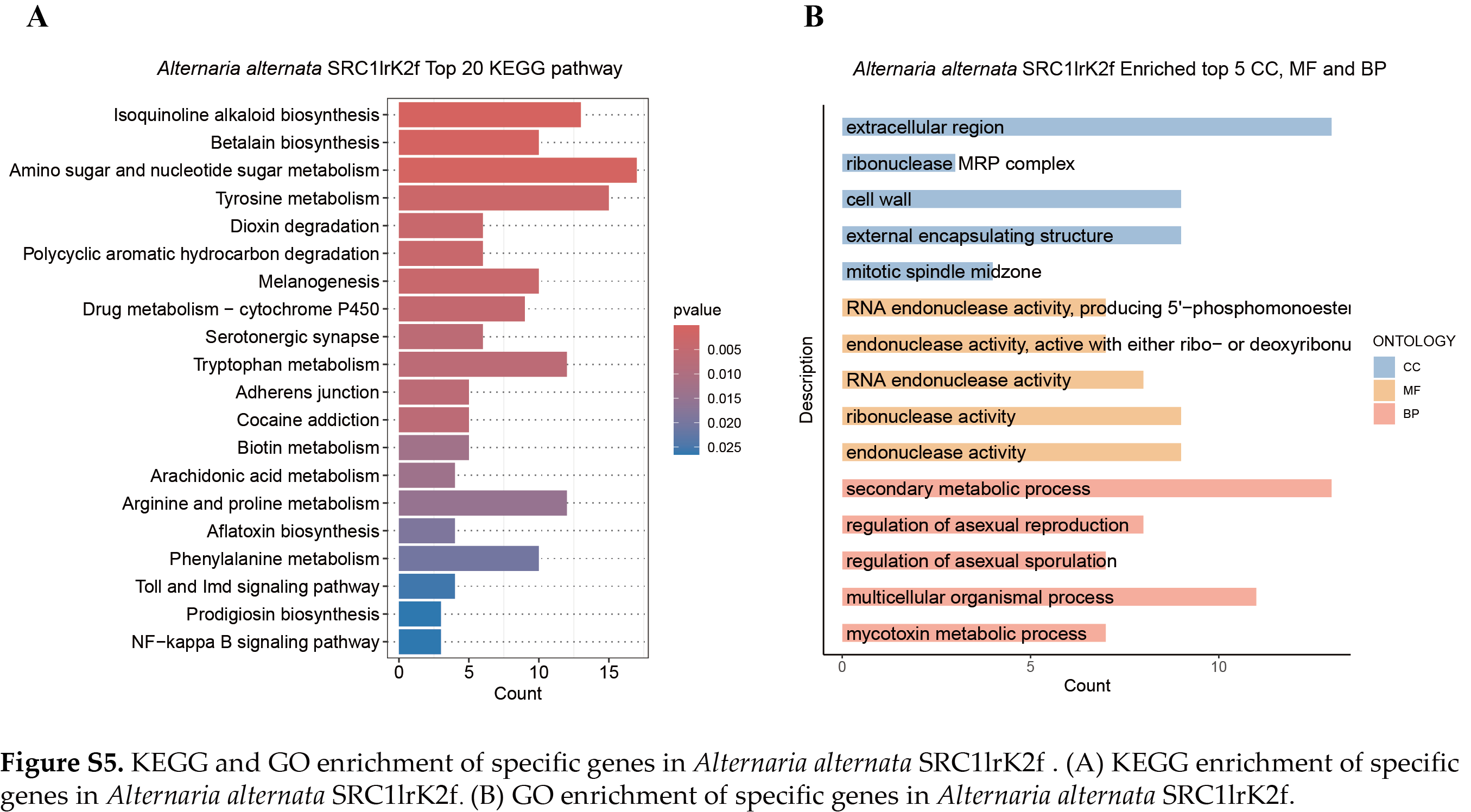

Supplement: Supplementary file 1 [file jof-11-00227-s001.zip › Supplementary/Figure S5.png]

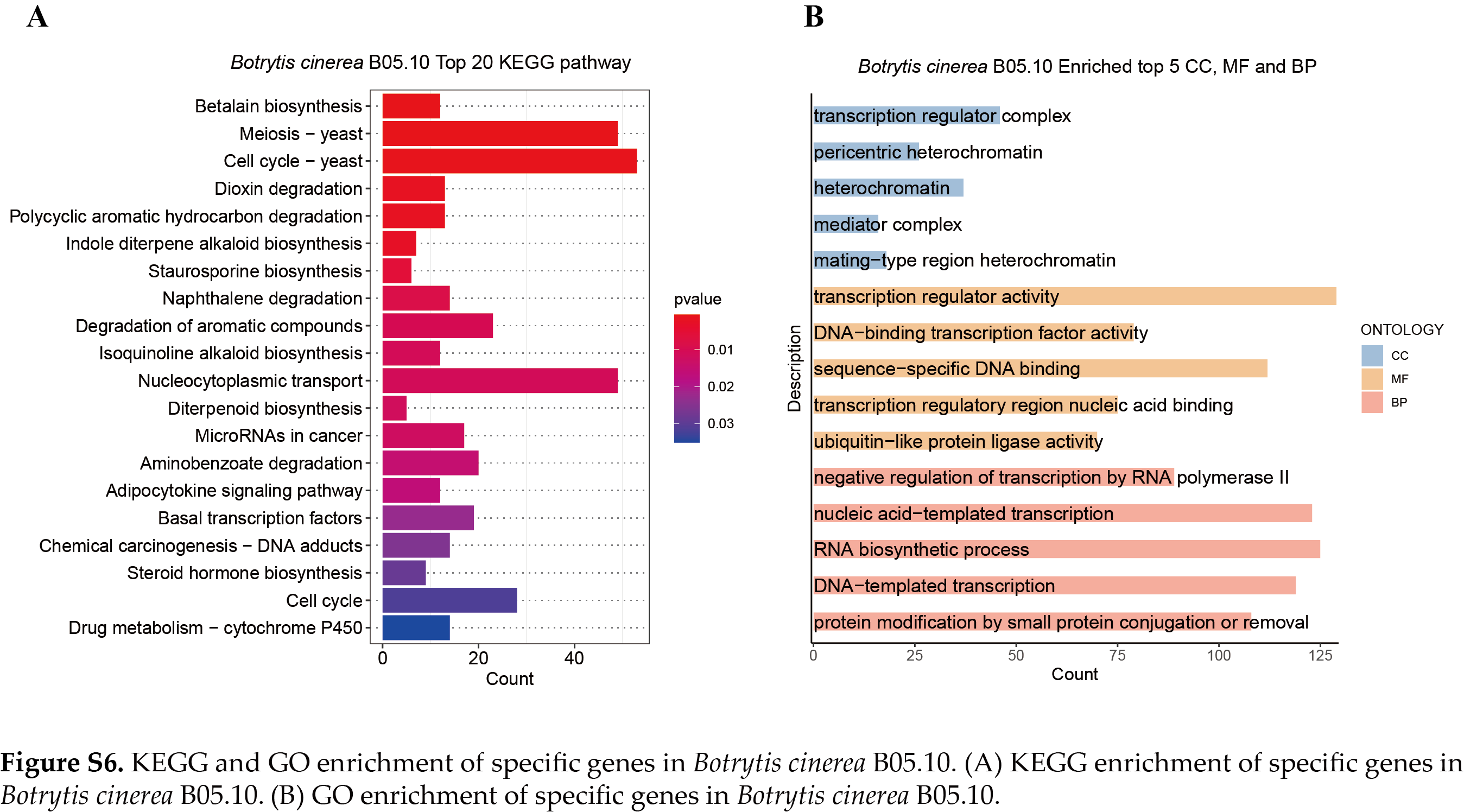

Supplement: Supplementary file 1 [file jof-11-00227-s001.zip › Supplementary/Figure S6.png]

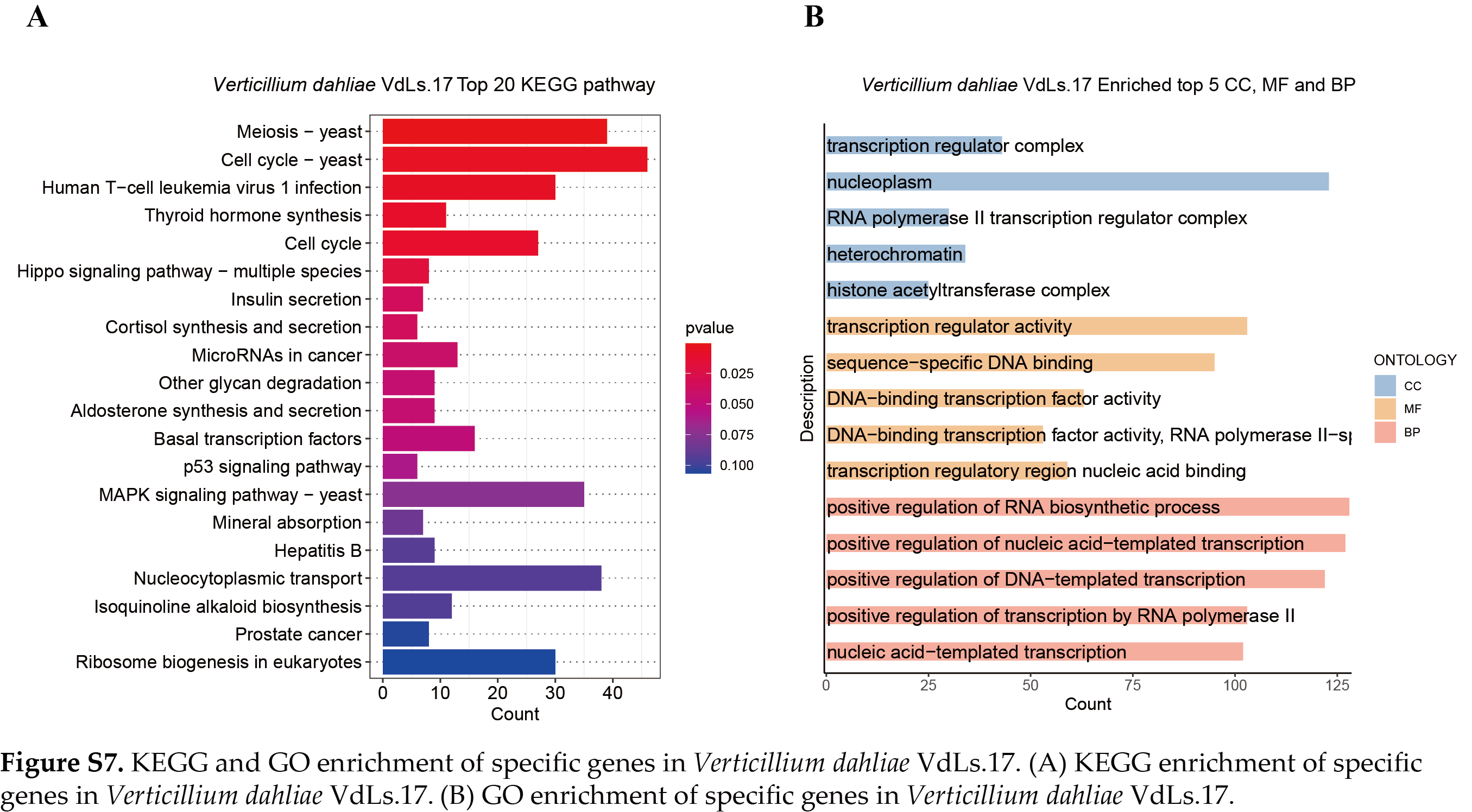

Supplement: Supplementary file 1 [file jof-11-00227-s001.zip › Supplementary/Figure S7.png]

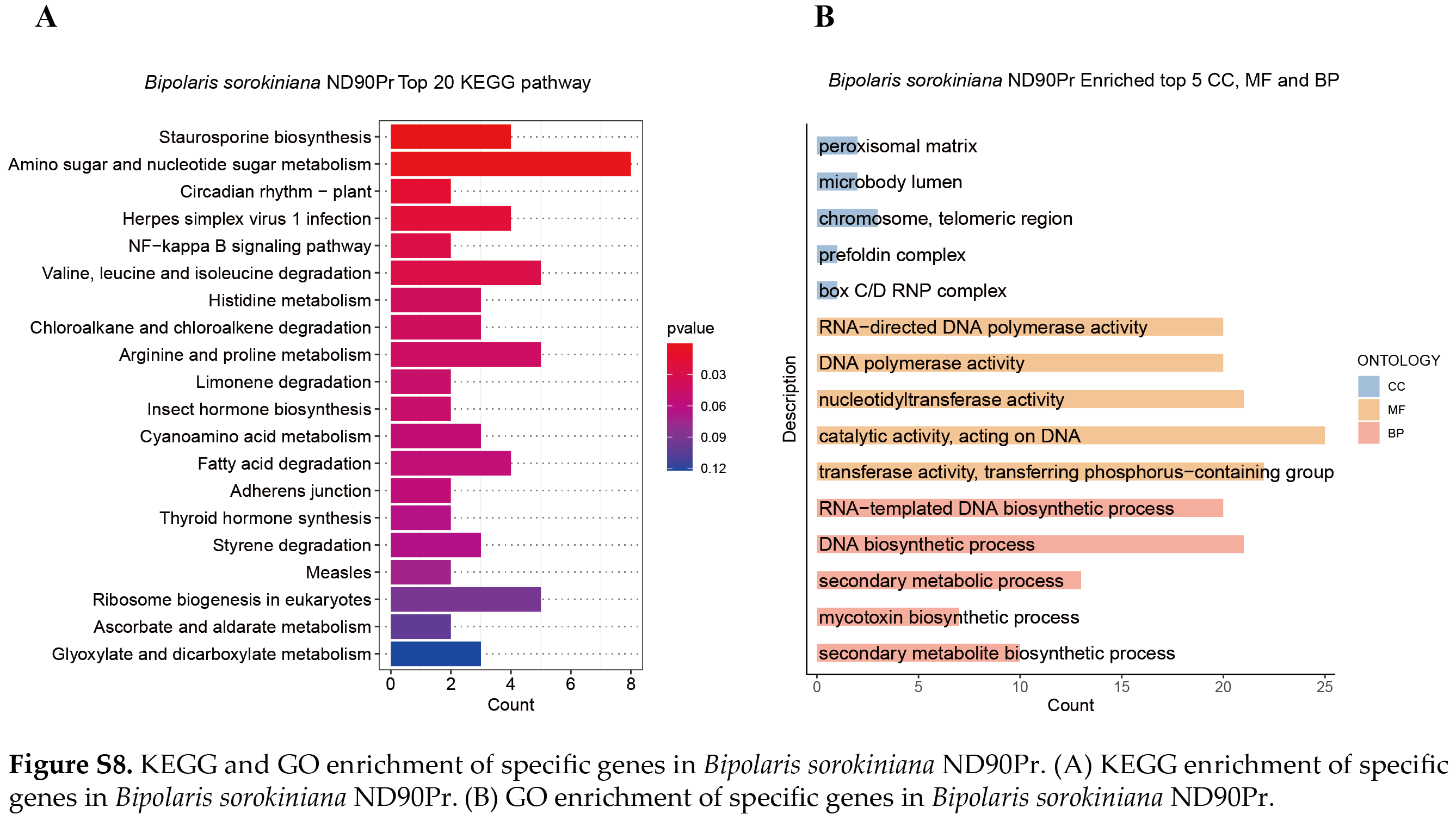

Supplement: Supplementary file 1 [file jof-11-00227-s001.zip › Supplementary/Figure S8.png]

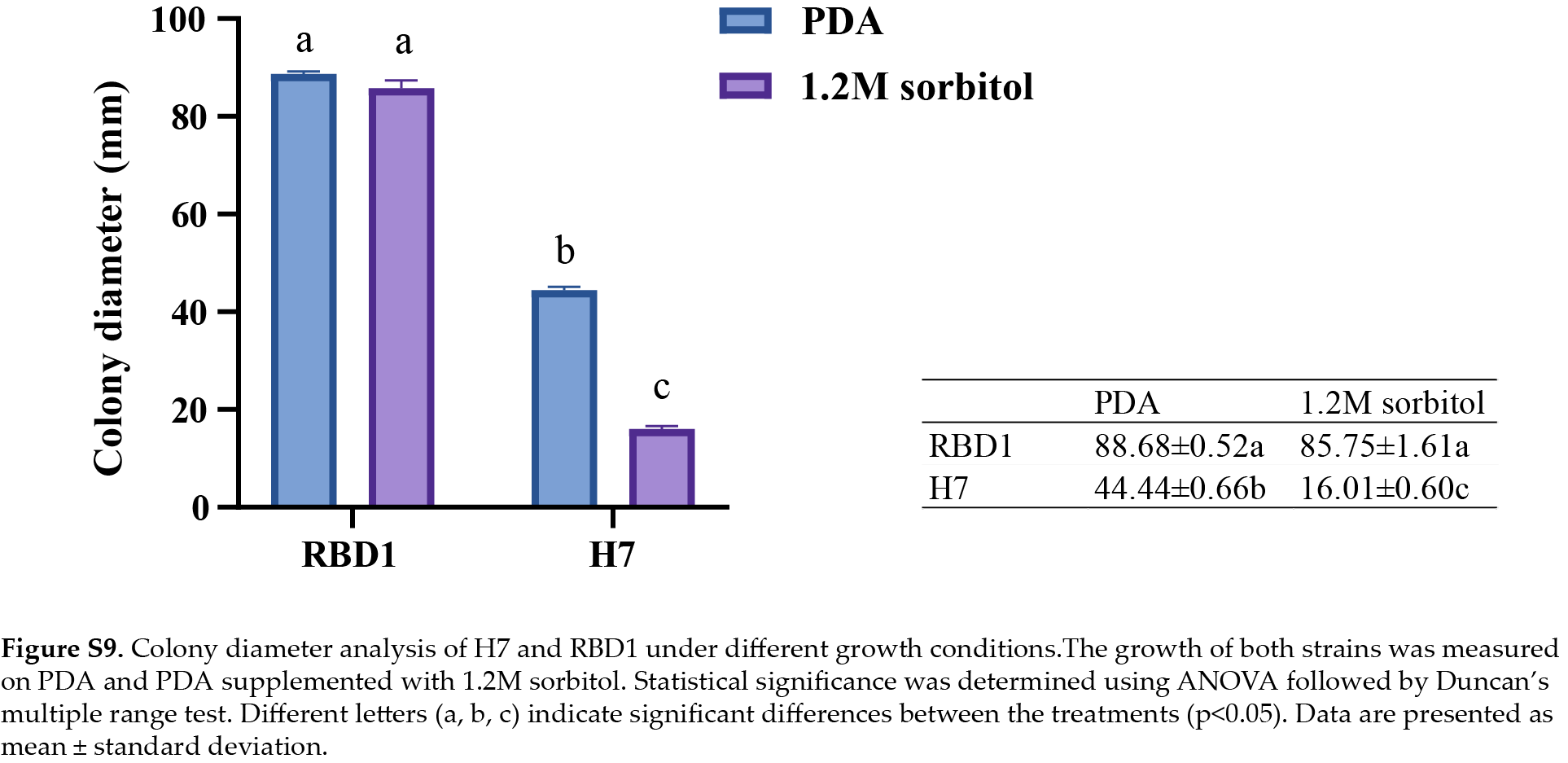

Supplement: Supplementary file 1 [file jof-11-00227-s001.zip › Supplementary/Figure S9.png]
